# New threats to health data privacy

**DOI:** 10.1186/1471-2105-12-S12-S7

**Published:** 2011-11-24

**Authors:** Fengjun Li, Xukai Zou, Peng Liu, Jake Y Chen

**Affiliations:** 1 Department of EECS, The University of Kansas, Lawrence, Kansas, USA; 2 Department of Computer and Information Science, IUPUI, Indianapolis, Indiana, USA; 3 College of IST, The Pennsylvania State University, University Park, Pennsylvania, USA

## Abstract

**Background:**

Along with the rapid digitalization of health data (e.g. Electronic Health Records), there is an increasing concern on maintaining data privacy while garnering the benefits, especially when the data are required to be published for secondary use. Most of the current research on protecting health data privacy is centered around data de-identification and data anonymization, which removes the identifiable information from the published health data to prevent an adversary from reasoning about the privacy of the patients. However, published health data is not the only source that the adversaries can count on: with a large amount of information that people voluntarily share on the Web, sophisticated attacks that join disparate information pieces from multiple sources against health data privacy become practical. Limited efforts have been devoted to studying these attacks yet.

**Results:**

We study how patient privacy could be compromised with the help of today’s information technologies. In particular, we show that private healthcare information could be collected by aggregating and associating disparate pieces of information from multiple online data sources including online social networks, public records and search engine results. We demonstrate a real-world case study to show user identity and privacy are highly vulnerable to the attribution, inference and aggregation attacks. We also show that people are highly identifiable to adversaries even with inaccurate information pieces about the target, with real data analysis.

**Conclusion:**

We claim that too much information has been made available electronic and available online that people are very vulnerable without effective privacy protection.

## Background

In recent years, a large amount of health data has been digitalized to reduce the cost and improve health care quality and efficiency. In various care delivering settings, patient health information such as demographics, problems, medications, progress notes, laboratory data, and medical history are recorded in the format of Electronic Health Records (EHRs). By 2009, 43.9% of the U.S. medical offices have adopted full or partial EHR systems [1]. The availability and legibility of electronic records not only facilitate sharing information in care-related activities, but also reduce medical errors and service time. With the broad adoption of EHRs, security and privacy of the digitalized health data becomes extremely critical considering the medical data are highly sensitive to the patients.

What further complicates the issue is that with the large amount of health data being digitalized, there is always a demand to publish the data for more intelligent use. Immense volumes of EHRs are published every year for secondary use, such as medical research, public health, government management, and other healthcare related services [2]. A typical EHR consists of a set of identifier attributes (e.g. *name*, *SSN*), quasi-identifier attributes (e.g. *gender*, *zipcode*), and sensitive attributes (e.g. *diseases*)*.* To protect the privacy of record owners, EHRs need to be de-identified [3-6] or anonymized [7-10] before publishing.

The key to the adoption of EHR and other health information technologies is the security and privacy of the digitalized, highly sensitive medical data. Hence, security and privacy becomes an important and popular topic in healthcare informatics research. Current research on protecting patient privacy in healthcare information systems are centralized around the protection of EHR – that is to protect patient information from being *abused* by authorized users, or being *accessed* by unauthorized outsiders, or being *re-identified* from health data published for secondary use. Regulations on data disclosure are set and legislated to address the threat from insider malfeasance and enhance the protection of health related data [11]. In the meantime, information technologies such as access control, encryption, file integrity check, firewalls, and anti-virus mechanisms defend against both internal abuse of and unauthorized access to the electronic health data. Various data de-identification [3-6] and data anonymization [7-10] techniques have also been proposed to sanitize the data sets in publishing and ensure that the data does not disclose any information about the sensitive data.

However, as the Web gains its popularity and touches many aspects of our daily life, it becomes the largest open-access source of personal information. The adversaries possess powerful weapons and rich knowledge, which are somehow provided by the victims themselves and are truly beyond the assumptions in the research literature.

First, large amount of public records have been made accessible online, including phone books, voter registration, birth/death records, etc. Although some of them enforce certain restrictions to defend against abusers, it is still relatively easy or inexpensive to crawl/download such records. More recently, online social network sites such as Facebook and MySpace have emerged to successfully attract a huge number of users, who willingly put their personal information to online social network sites to share with people. Second, with the new sophistication of information retrieval techniques and the advances of searching techniques in search engines, it becomes unexpectedly easy to conduct Web-scale extraction of users’ personal information that is readily available in various online social networks (e.g., [12-16]). As a result, malicious or curious adversaries could easily take advantage of these techniques to collect others’ private information, which is readily available from online public records or various social networks.

Therefore, it is reasonable for us to raise the question: *“when an attacker possesses a small amount of (possibly inaccurate) information from healthcare-related sources, and associate such information with publicly-accessible information from online sources, how likely the attacker would be able to discover the identity of the targeted patient, and what the potential privacy risks are.”*

To take a first step in answering this broad question, we study: (1) how user information from multiple online sources could be associated and utilized to compromise user privacy; (2) how user identity could be identified by comparing approximate information with public databases.

## Results and discussion

### Attacks on healthcare records

One effective protection on published EHR is data de-identification and anonymization. However, even with the sanitized data, sensitive attributes that pertain to an individual may be learned from other non-sensitive attributes in combination with external knowledge (e.g. voter registration list, phone books, etc.). The risks of such re-identification attacks have been intensively studied, which shows that the amount and types of an attacker’s external knowledge play an important role in reasoning about privacy in data publishing [9,10,17,18]. However, it is not easy, if not impossible, for a data publisher to know upfront what external knowledge the attacker possesses. Therefore, current research on privacy-preserving data publishing studies the problem from a theoretical perspective by making assumptions on attacker’s background knowledge, quantifying external knowledge regardless of its content, and sanitizing the data to ensure the amount of disclosure is below a specified threshold [17,18]. As a result, such protection, on one hand, does not take into account that large amount of external knowledge are accessible to the adversaries from various online sources (e.g. social networks), on the other hand, it might greatly distort the data and its secondary usages. Therefore, we believe it is of great importance to investigate the types and amounts of external knowledge that a powerful attacker possesses or infers from the immense volume of electronic data from *multiple online resources.* It not only provides evidence for efficient and optimal data sanitization, but also raises public concerns and awareness on the severeness of privacy threats and calls for effective protection.

Another potential privacy attack relates to the issue of insider misfeasance of sensitive medical data. Health care delivery personnel may violate privacy rules by disclosing or stealing private healthcare records for unauthorized usages, as depicted in [19]. This is a typical abuse/infraction with authorized data access. More often, the attackers do not have authorization for data access. They either eavesdrop or wiretap private information in transit or penetrate into EHR systems to get control of valuable health data. However, such types of attacks are often underestimated [20]. We believe such underestimation is partially from a fundamental misunderstanding that information revealed by carelessness or misuse is only one piece of the big picture and will not cause severe privacy disclosure. In this article, we will elaborate the severeness of such type of attacks in current information-rich context with an intuitive example.

### Attacks from external sources

Online social networks (OSNs) have become extremely popular in recent years. Users of ONSs often voluntarily disclose their personal information with surprising details. For example, Linked In users list their educational and working experiences to seek for potential career opportunities, and MedHelp users share details about their life and medical experiences expecting to receive pertinent medical suggestions from others. While releasing privacy-related information online, a fundamental misunderstanding of these information owners is that it is unlikely to link information pertaining to the same individual from different online sources. Unfortunately, with the advances of searching and information retrieval techniques, it is feasible for an attacker to *aggregate* personal information of a targeted user from different online sources, by associating unprotected but identifiable or semi-identifiable attributes (e.g. identical account names or email address of a careless user) [21]. Meanwhile, with governmental and industrial efforts, a large amount of public records have been digitalized and made available online. Most of them are indexed by commercial search engines with free access, while the others only require a minimum subscription fee to obtain full access. Adversaries could easily access and utilize such information to compromise others’ privacy, especially, their highly sensitive healthcare data.

From our real-world case study, we find it is highly possible for an attacker to aggregate disparate pieces of information from multiple (possibly medical-related) online sources, and associate the attributes to identify a targeted patient with high confidence.

### Real-world case study

**Figure 1:**
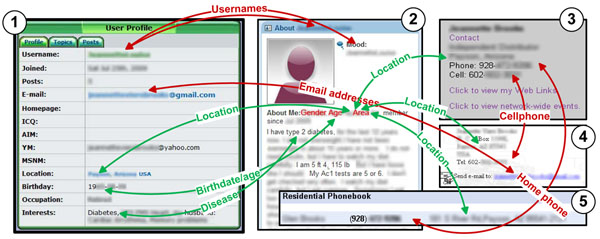
**A real-world example of cross-site information aggregation.** The target patient “Jean” has profiles on two online medical social networking sites (1) and (2). By comparing the attributes from both profiles, the adversary can link the two with high confidence. Furthermore, the attacker can use the attribute values to get more profiles of the target through searching the Web (3) and other online public data sets (4 and 5). By aggregating and associating the five profiles, Jean’s full name, date of birth, husband’s name, home address, home phone number, cell phone number, two email addresses, occupation, medical information including lab test results are disclosed unintendedly.

Figure 1 demonstrates an example from a real-world case study with a simulated attacker: “Jean” (whose full name has been discovered but removed here for privacy protection) has type II diabetes, so she actively participates in two online medicare social network sites, MedHelp (http://www.medhelp.org) and MP and Th1 Discussion Forum (http://www.curemyth1.org). Assume these are the only two “trivial” facts that the attacker knows about Jean: Jean has diabetes II, and Jean has profiles in both OSNs.

Since the user profiles in both OSNs are publicly accessible (after registration), the attacker’s first move is to crawl them from both sites, which is not an impractical task even for attackers with limited resource and computing power. Next, the attacker analyzes the crawled profiles to associate the profiles with matching attributes. The association should be conducted under two assumptions: (1) an individual is identifiable by an attribute set, and (2) the values of attributes in such attribute set are authentic information pertaining to an individual.

The second assumption is valid considering the nature of online medical social networks. The primary goal of online medical social networks is to serve as platforms for doctors and patients to discuss symptoms, compare treatment options and exchange medical advances. Therefore, in order to receive unbiased medical advices, the users often provide real and accurate personal information for most of the fields in user profiles, although they do use pseudonyms to register with the OSNs for privacy protection purpose. Hence, it is reasonable to believe the values of non-identifiable attributes in user profiles are real.

To validate the first assumption, we look at the files of the user profiles of both medical social network sites:

• MedHelp user profile includes *user name*, *gender*, *age*, *location*, *time joined*, *interest*, and a text field that allows more detailed inputs about the user and his/her particular medical conditions and problems.

• MP and Th1 user profile contains three types of fields: (1) fields directly related to an user’s activities in this site (e.g. *user name*, *joined time*)*;* (2) fields about his/her other online contacts (e.g. *email*, *homepage*, *ICQ*), and (3) fields about his/her personal information (e.g. *birthdate*, *occupation*, *location*, and a *text* field for specifying personal interests on medical information).

Both profiles contain the attribute set {Gender, Location, Age}. According to a famous study [22] of 1990 US census data, 87% of the U.S. population is uniquely identifiable by the attribute set {Gender, ZIP code, Birthdate}, and 53% of the U.S. population is uniquely identified by the attribute set {Gender, Location, Birthdate}, where the “location” refers to the city, town, or municipality in which the person resides. Since the two social networks target people with particular interests, it is reasonable to consider {Gender, Location, Age} as a quasi-identifier in this case.

From the crawled data, the attacker finds two “linkable” profiles, as shown in Figure 1(1) and Figure 1(2):

1. Profile 1 shows “my husband” that indicates the owner is a female, which is consistent with the gender shown in Profile 2;

2. The locations in both profiles are the same small town with approximately 15K population;

3. The birthdate in Profile 1 is consistent with the age shown in Profile 2.

In addition, the pseudo user names in both profiles are identical (and relatively unique), and both profiles demonstrate interests on a same disease and symptoms – diabetes type II. It is reasonable to link the two profiles at a certain confidence level and associate the attributes from both profiles to the same individual in the real-world – Jean. After that, more private attributes of Jean (e.g. times of doctor visit, diagnoses, prescriptions and medicines) are extracted from her postings on the two medical social network sites and added to the reconstructed profile of Jean.

From this baseline, we continue to further explore Jean’s private information from the Web.

• Explore by Email: Email addresses can effectively serve as unique identifiers. They generally provide helpful hint to link two profiles. In this example, with the email address provided in Profile 1, we retrieved Profile 4 (as shown in Figure 1(4)) through Web search engines. Profile 4 includes a phone number (which is found to be a cell phone number in later analysis) and a P.O. Box address. Both the phone number and the address indicate the same city as shown in Profiles 1 and 2.

• Explore by Phone Number: Phone numbers are commonly considered as unique identifiers. With the phone number from Profile 4, we further discovered Profile 3 (as shown in Figure 1(3)), which is a job-related page containing Jean’s cell and home phone numbers. Profiles 3 and 4 both contain the full name of “Jean”, and also provide a good hint on her occupation. Finally, with the home phone number, we also located Jean’s record in the residential phone book, which shows her husband’s name and their full home address (as shown in Figure 1(5)).

Finally, in this simulated attack, we successfully acquire five profiles, which highly likely belong to Jean, from both online social networking sites and publicly accessible online data. By associating the five profiles, we have recovered types of private information about Jean, including her full name, date of birth, husband’s name, home address, home phone number, cell phone number, two email addresses, occupation and medical information including lab test results.

On the other hand, even without Profiles 3, 4 and 5, an attacker could also utilize public records to get more information about Jean: with the attribute set {gender, birthday, location}, Jean’s identity (e.g. full name, address, and phone number) is recoverable from public birth records, voters registration records or online phone books. With her full name, more information about Jean is subsequently discovered from various social networks. Finally, when Jean’s hospital publishes de-identified patient records to support medical research, the attacker with external knowledge obtained from above process is highly likely to re-identify Jean’s record.

The example reveals a serious privacy issue in both social networks and healthcare informatics. The entire process includes three steps: *attribution*, *inference*, and *aggregation* attacks. In attribution, identifiable, semi-identifiable or sensitive attributes are learned/extracted from various sources over the web. Particularly, three types of online resources are considered in the example: (1) public-accessible online databases: voters registration records, phone books, birth and death records, (2) online social network sites with explicit identifiable attributes (e.g. *LinkedIn*, *Facebook*, etc.) as well as specified healthcare-related social networks (e.g. *MedHelp*)*;* and (3) commercial search engines, which index a good portion of the web. In inference, more attributes are further discovered from social activities and relationships through statistical learning or logical reasoning. In aggregation, records retrieved from different sources that potentially pertain to the same individual are linked under strong or weak evidences, in which strong evidences include matching identifiers or quasi-identifiers, and weak evidences are similarities identified from a statistical perspective. As we have shown in the example, the attacks are very valid and do not require excessive resources or techniques. Therefore, people are very vulnerable under such attacks, if they do not carefully protect their online identities. A powerful privacy protection tool is expected to defend against such attacks.

### Attacks with approximate information

Besides privacy attacks against digitalized medical records and healthcare information systems, adversaries also seek to obtain valuable information with non-technical kind of intrusions such as insider incidents or social engineering. With a vague definition, insider incidents often involve abuses such as inside personnel accidental leaking or stealing information, using pirated software, or accessing questionable webpages. Social engineering relies on people’s unawareness of valuable information and carelessness in protection and becomes one of the major attacks towards user privacy. However, in most cases, information obtained from non-digital channels are not accurate due to the difficulty of accessing information, human capabilities or errors. For example, in today’s medicine practice, many doctors record patients’ medical information (e.g. symptoms, diagnoses, prescriptions, etc) with a audio recorder, and hire external companies to convert recordings into digital records. In the process, an adversary may steal the recording and learn detailed medical conditions of a patient, however, he may learn inaccurate information about patient’s identity (e.g. he may not be able to get the correct spelling of the patient’s name from doctor’s voice). One may assume that the inaccuracy of attackers’ knowledge may bring difficulty for them to compromise user identity or privacy. Unfortunately, such inaccuracy could be corrected by collaborating with external information sources, and the privacy risks causes by such attacks should no longer be ignored.

Here is a simple but representative example: Dr. Bob treats Alice in the hospital, while Malory eavesdrops the conversation, or peeps the record. Malory possesses the full prescription with an inaccurate version of Alice’s last name (due to Dr. Bob’s squiggling handwriting). Mallory does not know Alice, so he starts his attack by first looking into the phonebook for all “similar” names in the neighborhood. The question is: *What is Malory’s opportunity of accurately recovering Alice’s full name*?

### k-approximate-anonymity

To further articulate this problem, we define *k-approximate-anonymity.*

**[k-approximate-anonymity]:** Given a data-set *D*, and a distance function *dist*(*r*_1_, *r*_2_) that returns the distance for any two records on the dataset; for any record *r*, if there exists *k* – 1 records *r_x_* that *dist*(*r*, *r_x_*) <= *l* where *l* is a preset threshold, we conclude that *D* satisfies *k-*approximate-anonymity or *k*-*l*-anonymity with *dist.*

In the above definition, when *l* = 0, it becomes the original *k-anonymity.* It basically says that when Mallory possesses approximate information on a target, he cannot distinguish the target from *k* – 1 other records in the database.

**Figure 2:**
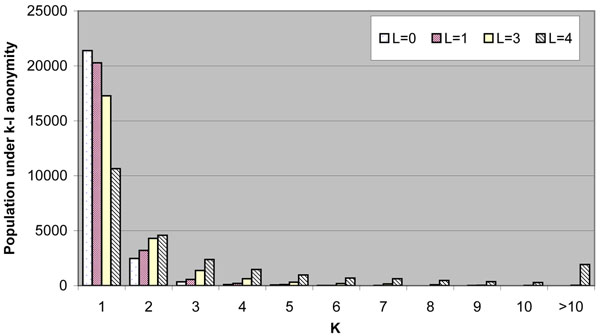
**Population under k-l-anonymity.** This example measures the distinguishness of the population under k-l-anonymity, using the first name as the identifier. The histogram plots the number of individuals whose first names differ with other (*k*-1) records for *l* letters. It shows within 24,399 records, most of them (more than 70% as shown in the first bar with *k*=1) are quite distinguishable.

To simulate the above scenario, we have designed an experiment to study the identifiability of real names in the presence of inaccurate information from the attackers. We first implement a crawler to download the public residential phone book. In a few days, it successfully collects 24,399 records from State College area, which covers approximately 64% of the population (according to 2000 census data). In each record, we have phone number, first and last names, and full residential address. In the experiments, we use full name as identifiers, and use the Levenshtein distance (edit distance) [23] as the distance function. For different threshold *l*, we show the population whose names are protected under *k-l-anonymity* in Figure 2.

From the figure, we can see that, with larger *l*, people are less identifiable with their names. However, overall, most (more than 70%) people are uniquely identifiable even when *l*=2. It means that even though Mallory gets an inaccurate name of the target, he has a good chance to correct the mistake and limit the target to a small range with the help of digital phonebooks. Even when Mallory gets four letters wrong in the name, in more than 80% of the cases, his target is limited to no more than 5 candidates, i.e., he only needs to further examine no more than 5 records to identify the target. As we expected, people with longer names or unusual names are more vulnerable, while people with shorter or more popular names are less identifiable, especially when the attacker possesses inaccurate information.

## Conclusions

In this work, we study the privacy vulnerabilities when medical records join with the Web. First, we show that multiple information sources (e.g. social networks and public records) could be utilized by the attackers. With attribution, inference and aggregation attacks, the attacks are capable of reconstructing very comprehensive user profiles, with various types of highly sensitive and private information (e.g. names, phone numbers, birth dates, diseases, lab test results, etc). On the other hand, we show that people are very identifiable if the attackers are equipped with information retrieval and data mining techniques. Even though an attacker only possesses a piece of inaccurate information, he is still highly likely to identify the target with the help of external information sources.

## Competing interests

The authors declare that they have no competing interests.

## Authors' contributions

FL conceived the study and all the authors designed it. FL performed experiments and statistical analysis, and drafted the manuscript. JYC and XZ helped with the conception and design of the statistical analysis model. PL participated in the design and coordination of the project. All authors have read and approved the final manuscript.
